# Medicaid enrollment among previously uninsured Americans and associated outcomes by race/ethnicity—United States, 2008‐2014

**DOI:** 10.1111/1475-6773.13085

**Published:** 2018-11-05

**Authors:** Tyler N. A. Winkelman, Joel E. Segel, Matthew M. Davis

**Affiliations:** ^1^ Division of General Internal Medicine Hennepin Healthcare Minneapolis Minnesota; ^2^ Hennepin Healthcare Research Institute Minneapolis Minnesota; ^3^ Department of Health Policy and Administration The Pennsylvania State University University Park Pennsylvania; ^4^ Penn State Cancer Institute The Pennsylvania State University Hershey Pennsylvania; ^5^ Mary Ann & J. Milburn Smith Child Health Research Program Ann and Robert H. Lurie Children's Hospital Chicago Illinois; ^6^ Department of Pediatrics Northwestern University Feinberg School of Medicine Chicago Illinois

**Keywords:** access to care, disparities, health care costs, medicaid, uninsured

## Abstract

**Objectives:**

To examine the person‐level impact of Medicaid enrollment on costs, utilization, access, and health across previously uninsured racial/ethnic groups.

**Data Source:**

Medical Expenditure Panel Survey, 2008‐2014.

**Study Design:**

We pooled multiple 2‐year waves of data to examine the direct impact of Medicaid enrollment among uninsured Americans. We compared changes in outcomes among nonpregnant, uninsured individuals who gained Medicaid (N = 963) to those who remained uninsured (N = 9784) using a difference‐in‐differences analysis.

**Principal Findings:**

Medicaid enrollment was associated with significant increases in total health care costs and total prescription drug costs and a significant decrease in out‐of‐pocket costs. Among those who gained Medicaid, prescription drug use increased significantly relative to those who remained uninsured. Medicaid enrollment was also associated with a significant increase in reporting a usual source of care, a decrease in foregone care, and significant improvements in severe psychological distress. Changes in total prescription drug costs and total prescription drug fills differed significantly across each racial/ethnic group.

**Conclusions:**

Among a national sample of uninsured individuals, Medicaid enrollment was associated with substantial favorable changes in out‐of‐pocket costs, prescription drug use, and access to care. Our findings suggest Medicaid is an important tool to reduce insurance‐related disparities among Americans.

## 1 INTRODUCTION

Enrollment in Medicaid, a government‐sponsored program that covers health care costs for low‐income individuals and families in the United States, increased from 35 million to over 74 million between 2000 and 2017.^1^, ^2^ Implementation of two policies during this period, the Health Insurance Flexibility and Accountability initiative and the Affordable Care Act (ACA), significantly increased Medicaid enrollment in some states and not others, creating natural experiments to study the population‐level impact of Medicaid.^3^, ^4^, ^5^, ^6^, ^7^ A large body of evidence stemming from these experiments suggests Medicaid has positive effects on access to care, health, and financial security.^8^, ^9^, ^10^, ^11^, ^12^, ^13^, ^14^ For example, Medicaid expansion under the ACA led to an 8.2 percentage point improvement in insurance coverage,^5^ a 12.1 percentage point increase in access to primary care,^15^ a 3.4 percentage point decrease in self‐reported lifetime depression diagnoses among individuals with chronic conditions,^8^ and a decrease in unpaid medical bills of $3.4 billion over 2 years.^10^

Although the population‐level effects of state Medicaid expansions (i.e, average treatment effects) are well documented, less is known about Medicaid's direct impact among people who gain Medicaid after a period of uninsurance (i.e, average treatment effect on the treated). The Oregon Health Insurance Experiment (OHIE), the most rigorous study to date to examine the impact of gaining Medicaid at the individual level, found that uninsured individuals who gained Medicaid in Oregon state had significantly lower levels of depression and out‐of‐pocket spending and higher levels of prescription medication use than individuals who were not enrolled in Medicaid.^16^, ^17^, ^18^ No other contemporary studies have followed individuals who gain Medicaid after a period of uninsurance. Such studies would be helpful to build on the findings of the OHIE and may shed light on whether identified associations are consistent across time, region, and race/ethnicity. These data are critical because they can inform ongoing policy debates regarding the design and funding of Medicaid, as well as efforts to improve racial and ethnic disparities in care.^12^, ^19^, ^20^, ^21^, ^22^

We used a nationally representative panel survey to examine the impact of Medicaid enrollment on disparities in health care costs, access to care, and general health measures among previously uninsured Americans who transitioned onto Medicaid and stratified our analyses by race/ethnicity. Based on findings from the OHIE and population‐level studies, we hypothesized that Medicaid enrollment would be associated with lower out‐of‐pocket costs, higher levels of prescription medication use and usual sources of care, and improvements in mental health.

## 2 METHODS

### 2.1 Data and study population

We used 2008‐2014 Medical Expenditure Panel Survey (MEPS) data. Medical Expenditure Panel Survey is a nationally representative survey that compiles demographic, health insurance, health care costs, utilization, and access, and self‐reported health data. Medical Expenditure Panel Survey has an overlapping panel design that surveys each respondent five times over a period of 2 years. Therefore, in any given year, half the sample is in their first year and half in their second year. To create our analytic sample, we restricted analyses to respondents who had 2 years of data, were between the ages of 19 and 64 (inclusive) in their first year of MEPS, who were not pregnant in either year, and whose family income was ≤400 percent of the federal poverty level in each year. We excluded pregnant individuals because pregnancy is a categorical eligibility for Medicaid and because patterns of health care in pregnancy are substantively different than for other health circumstances.

Our sample consisted of two groups: (a) those who remained uninsured throughout the 2‐year study period and (b) those who gained Medicaid after a period of uninsurance. We defined the latter population as respondents who were uninsured for at least 6 months within their first 9 months in MEPS (Period 1) and had at least 6 months of Medicaid coverage for the remaining 15 months (Period 2). We chose to set our cut‐point at 9 months because nearly all individuals who gained Medicaid in our sample would have completed two rounds of surveys while uninsured prior to the fourth quarter of their first year in MEPS, and because this definition is similar to other evaluations of low‐income populations who gain Medicaid.^23^ To ensure all outcomes derived from round 2 of MEPS occurred in the first 9 months, we excluded individuals who did not complete round 2 by September of their first year in MEPS. Additionally, in sensitivity analyses, we vary each group definition to test the robustness of our results.

### 2.2 Outcome measures

Health care costs, health care utilization, and self‐reported general and mental health were obtained in each of the five MEPS survey rounds, but due to how the sample was created, we did not include values from round 3. We used values from rounds 1 and 2 during Period 1 and rounds 4 and 5 during Period 2 to ensure similar follow‐up time across each period and to allow for a brief washout period between uninsurance and Medicaid enrollment. Access measures and psychological distress were only reported in rounds 2 and 4.

#### 2.2.1 Health care costs

We examined total health care costs and total out‐of‐pocket costs for individuals in Period 1 and Period 2, as well as total and out‐of‐pocket prescription drug costs. Each cost measure was adjusted to 2014 dollars using the Medical Component of the Consumer Price Index.^24^ For inpatient, outpatient, and emergency department (ED) visits and prescription drug costs, MEPS collects data from the participating individual and their medical providers.^25^

#### 2.2.2 Health care utilization

We estimated having any ED visit, total number of ED visits per person, any inpatient visit, total number of inpatient visits per person, any prescription drug fill, and total number of prescription drug fills per person. These were obtained through medical provider records.

#### 2.2.3 Health care access

Several health care access measures were assessed, including a usual source of care, foregone medical care (i.e, “unable to get medical care, tests, or treatments a respondent or a doctor believed to be necessary”), delayed medical care (i.e, “delayed medical care, tests, or treatments a respondent or a doctor believed to be necessary”), or unable to get necessary prescription drugs (i.e, “unable to obtain prescription medicines a respondent or a doctor believed to be necessary”). Each of these outcomes was asked in rounds 2 and 4 and refers to the preceding 12 months.

#### 2.2.4 Health outcomes

Our final outcome measures included several self‐reported health measures: general health (fair or poor health in any survey round for each period), mental health (fair or poor mental health in any survey round for each period), and severe psychological distress (i.e, Kessler index score of 13 or greater).^26^

### 2.3 Covariates

We considered several sociodemographic characteristics that are known to be associated with health insurance status.^6^, ^12^ In our multivariable linear regression models, we controlled for age, sex, race/ethnicity, marital status, education, region, family size and income, calendar year, and number of chronic conditions at baseline. For purposes of subgroup analysis, we created mutually exclusive racial/ethnic groups: White, non‐Hispanic; Black, non‐Hispanic; Hispanic; and Other race, non‐Hispanic. Chronic conditions were identified in Period 1 if a respondent reported ever being diagnosed with arthritis, asthma, high blood pressure, cancer, heart disease (i.e, any report of angina, coronary heart disease, myocardial infarction, or other heart disease), high cholesterol, diabetes, emphysema, or stroke and then summed to create a count of chronic conditions. These classifications are generally consistent with US Department of Health and Human Services’ recommendations for standardization of chronic condition identification.^27^

### 2.4 Statistical analysis

We first examined whether there were differences between individuals who gained Medicaid and those who remained uninsured by comparing means of baseline sociodemographic characteristics.

Next, for each of our outcomes, we compared baseline values (i.e, in Period 1) to follow‐up values (i.e, in Period 2) for individuals who gained Medicaid vs for those who remained uninsured. We also stratified our analysis of Medicaid gainers by race/ethnicity. Due to considerable heterogeneity and low sample size within “Other, non‐Hispanic,” we excluded this group in stratified analyses. Significance testing of outcome differences between Period 1 and Period 2 was conducted using multivariable linear regression, incorporating the characteristics identified above.

In our final set of analyses, we estimated multivariable linear regression models to assess how gaining Medicaid affected each of our four sets of outcomes relative to remaining uninsured. In each model, we used a difference‐in‐differences framework, interacting time period with an indicator of Medicaid enrollment, to compare changes in the outcomes for Medicaid gainers to changes among those who remained uninsured between Period 1 and Period 2. Analyses were conducted among the entire sample and also stratified by race/ethnicity. We accounted for the complex survey design in MEPS using svy commands in Stata 14.2 (StataCorp, College Station, TX, USA) for all analyses.

In addition to our primary multivariable regression specifications, we ran a series of sensitivity analyses to examine the robustness of our results. First, we compared linear trends for costs, utilization, and self‐reported health outcomes in Period 1 among individuals who gained Medicaid and those who remained uninsured. We did not assess Period 1 trends for access measures because data for these outcomes were only collected once during Period 1. Second, to ensure those who gained Medicaid and those who remained uninsured were well matched, we reestimated our difference‐in‐differences regressions using entropy balancing, an approach that directly reweights the control group to match the means (or other moments) of the treatment group.^28^, ^29^, ^30^ We estimated two models using entropy balancing, first weighting with the covariates used in our baseline approach and second, weighting with round 1 and 2 values of the outcomes, using costs when the outcomes were not measured more than once in a period. In both cases, we used the resulting weights to estimate difference‐in‐differences regressions similar to our baseline analyses. Third, for cost variables, we re‐estimated the models using a two‐part model.^31^, ^32^, ^33^ Finally, we made a variety of modifications to our definitions of both the Medicaid gainer population and the control group, in each case varying the number of months they were either uninsured or had Medicaid coverage.

## 3 RESULTS

Our sample included 10 747 individuals, including 963 Medicaid gainers and 9784 individuals who remained uninsured in both years of their participation in MEPS. As illustrated in Table 1, those who gained Medicaid were more likely to be female, White, non‐Hispanic, Black, non‐Hispanic, below 100 percent of the federal poverty level, from the Midwest or Northeast, and more likely to be in the first cohort after ACA Medicaid expansion (i.e, their first year of MEPS participation was 2013).

**Table 1:** Sociodemographic characteristics of study population

Characteristic	Weighted % (95% CI)	
	Medicaid “Gainer” (N = 963)^a^	Remained uninsured (N = 9784)^a^
Weighted annual sample size	1.05 million	10.4 million
Age (y)	39.2 (38.1‐40.2)	38.0 (37.6‐38.4)
Female	53.7 (49.4‐58.0)	41.8 (40.6‐42.9)
Race/ethnicity		
White, non‐Hispanic	46.5 (41.1‐51.9)	40.4 (36.9‐43.9)
Black, non‐Hispanic	20.1 (16.7‐23.5)	14.2 (11.7‐16.7)
Hispanic	24.8 (20.8‐28.7)	39.1 (34.3‐43.9)
Other, non‐Hispanic	8.6 (5.8‐11.5)	6.3 (5.1‐7.4)
Married	30.1 (25.9‐34.3)	38.0 (35.5‐40.4)
Education		
Less than HS	26.2 (22.4‐30.0)	30.7 (28.8‐32.7)
HS	67.4 (63.2‐71.6)	61.0 (59.3‐62.8)
Bachelor's or higher	5.3 (3.4‐7.2)	7.1 (6.2‐8.0)
Family size (no.)	2.9 (2.8‐3.1)	3.2 (3.1‐3.3)
Income (% FPL)		
<100%	49.8 (45.3‐54.2)	32.3 (30.6‐34.0)
100%‐124%	10.6 (7.9‐13.2)	9.3 (8.4‐10.1)
125%‐199%	21.3 (18.1‐24.5)	27.7 (26.4‐29.0)
200%‐399%	18.4 (14.6‐22.2)	30.8 (28.9‐32.6)
Region		
Midwest	23.3 (18.7‐27.9)	16.1 (13.8‐18.4)
Northeast	20.6 (14.7‐26.4)	10.3 (8.8‐11.7)
South	27.4 (23.2‐31.5)	46.8 (42.8‐50.8)
West	28.8 (23.4‐34.2)	26.9 (24.1‐29.6)
Year of study entry^a^		
2008	14.5 (11.4‐17.7)	19.2 (18.1‐20.3)
2009	12.0 (9.3‐14.7)	16.2 (15.0‐17.5)
2010	14.1 (11.1‐17.1)	15.7 (14.6‐16.9)
2011	16.0 (12.2‐19.7)	18.0 (16.9‐19.2)
2012	17.0 (13.9‐20.2)	17.1 (16.0‐18.2)
2013	26.4 (22.2‐30.7)	13.7 (12.5‐14.9)
Chronic conditions (mean)	1.2 (1.1‐1.3)	0.7 (0.6‐0.7)

a Each participant was enrolled in the study for 2 y.

### 3.1 Health care costs

Next, we examined how each outcome changed from Period 1 to Period 2 among the entire sample. As indicated in Table 2, both populations experienced significant increases in total costs—an increase of over $1700 for Medicaid gainers compared to an increase of just over $100 for those remaining uninsured. Both populations also experienced significant increases in total prescription drug costs—a $621 increase for those gaining Medicaid and a $48 increase for those remaining uninsured. While those remaining uninsured saw small increases in out‐of‐pocket costs, those gaining Medicaid experienced decreases in both total out‐of‐pocket costs and total prescription drug out‐of‐pocket costs (decreases of $219 and $142, respectively).

**Table 2:** Changes in health care costs, utilization, access, and outcomes among nonelderly uninsured adults who gained Medicaid or remained uninsured

Outcome	Unadjusted estimates					
	Medicaid gainers (N = 963)			Remained uninsured (N = 9784)		
	Period 1	Period 2	*P* value^a^	Period 1	Period 2	*P* value^a^
Health care costs						
Total costs ($)^b^	1393	3109	<0.001	605	717	0.052
Out‐of‐pocket costs ($)^b^	418	199	0.02	210	240	0.12
Total prescription drug costs ($)^b^	432	1053	<0.001	146	194	<0.001
Out‐of‐pocket prescription drug costs ($)^b^	260	118	0.10	92	109	0.11
Health care utilization						
Any ED visit (%)^b^	18.2	21.1	0.27	8.8	8.8	0.96
ED visits/person^b^	0.27	0.33	0.15	0.12	0.12	0.51
Any inpatient visit (%)^b^	5.2	6.9	0.21	1.8	1.6	0.59
Inpatient visits/person^b^	0.07	0.09	0.20	0.02	0.02	0.93
Any prescription drug fill (%)^b^	50.1	60.8	<0.001	32.3	30.7	0.003
Prescription drug fills/person^b^	6.35	12.67	<0.001	2.86	3.64	<0.001
Health care access						
Have usual source of care (%)	55.5	70.1	<0.001	38.4	40.2	0.03
Unable to get necessary medical care (%)	18.3	10.3	<0.001	10.1	9.1	0.04
Delayed necessary medical care (%)	15.0	10.5	0.01	7.0	6.5	0.23
Unable to get necessary prescription (%)	11.7	8.1	0.050	5.5	4.7	0.01
Health outcomes						
Fair/poor health (%)^b^	39.0	36.7	0.30	23.4	21.0	<0.001
Fair/poor mental health (%)^b^	26.1	24.4	0.31	12.0	12.9	0.06
Severe psychological distress (%)	18.4	14.1	0.01	6.7	6.5	0.48

a Regression tests significance of increase between Period 1 and Period 2 while controlling for age, sex, race/ethnicity, marital status, education, region, family size, family income, year, and number of chronic conditions.

b Based on Medical Expenditure Panel Survey rounds 1 and 2 (Period 1) and rounds 3 and 4 (Period 2).

Among Medicaid gainers, White, non‐Hispanic and Hispanic individuals experienced significant increases in total costs, whereas total costs among Black, non‐Hispanic individuals did not increase significantly (Table 3). Increases in total prescription drug costs were statistically significant across racial/ethnic groups.

**Table 3:** Changes in health care costs, utilization, access, and outcomes among nonelderly uninsured adults who gained Medicaid by race/ethnicity

Outcome	Unadjusted estimates								
	White, non‐Hispanic (N = 260)			Black, non‐Hispanic (N = 267)			Hispanic (N = 362)		
	Period 1	Period 2	*P* value^a^	Period 1	Period 2	*P* value^a^	Period 1	Period 2	*P* value^a^
Health care costs									
Total costs ($)^b^	1816	4254	<0.001	1467	1888	0.31	899	2331	0.02
Out‐of‐pocket costs ($)^b^	647	299	0.07	302	73	0.06	184	125	0.18
Total prescription drug costs ($)^b^	696	1587	0.002	182	540	0.02	245	598	0.001
Out‐of‐pocket prescription drug costs ($)^b^	472	159	0.09	65	56	0.62	96	92	0.83
Health care utilization									
Any ED visit (%)^b^	23.3	23.4	0.99	15.6	18.0	0.47	14.0	17.8	0.40
ED visits/person^b^	0.36	0.40	0.65	0.19	0.24	0.29	0.19	0.28	0.16
Any inpatient visit (%)^b^	7.0	9.2	0.41	6.0	5.3	0.73	2.9	5.6	0.11
Inpatient visits/person^b^	0.08	0.11	0.39	0.08	0.07	0.64	0.04	0.10	0.22
Any prescription drug fill (%)^b^	62.6	68.0	0.21	40.1	56.4	<0.001	40.7	53.7	0.001
Prescription drug fills/person^b^	8.7	16.8	<0.001	4.9	10.9	0.002	4.7	8.0	<0.001
Health care access									
Have usual source of care (%)	61.7	76.5	<0.001	56.9	64.7	0.049	46.6	64.2	<0.001
Unable to get necessary medical care (%)	25.9	17.6	0.03	14.0	4.1	<0.001	12.0	3.1	0.001
Delayed necessary medical care (%)	24.2	16.3	0.03	8.9	5.6	0.14	6.6	3.9	0.34
Unable to get necessary prescription (%)	19.1	11.4	0.048	8.4	9.2	0.73	3.5	3.4	0.97
Health outcomes									
Fair/poor health (%)^b^	45.8	42.8	0.40	32.5	30.8	0.57	35.3	35.2	0.85
Fair/poor mental health (%)^b^	35.2	31.7	0.31	20.0	20.6	0.83	18.6	16.6	0.35
Severe psychological distress (%)	23.8	20.4	0.27	13.9	7.8	0.09	14.6	8.7	0.01

ED, emergency department.

Regression tests significance of increase between Period 1 and Period 2 while controlling for age, sex, race/ethnicity, marital status, education, region, family size, family income, year, and number of chronic conditions.

Based on Medical Expenditure Panel Survey rounds 1 and 2 (Period 1) and rounds 3 and 4 (Period 2).

### 3.2 Health care utilization

We found no significant changes in ED and inpatient visits among individuals who gained Medicaid compared to those who remained uninsured (Table 2). Those gaining Medicaid saw a significant 10.7 percentage point increase in having any prescription drug fills, while those remaining uninsured saw a modest, but statistically significant, decrease of 1.6 percentage points. However, both experienced significant increases in total number of prescription drug fills—an increase of over six fills for Medicaid gainers and a more modest increase of 0.8 fills for those remaining uninsured. Decreasing levels of any drug fill and increasing number of total drug fills among individuals who remain uninsured may be related to increasing resource use among a concentrated sample of uninsured individuals over time.^34^

Any prescription drug fills increased significantly among Black, non‐Hispanic and Hispanic individuals who gained Medicaid, but not White, non‐Hispanic individuals (Table 3). However, total prescription drug fills increased across each racial/ethnic group, with the largest gains among White, non‐Hispanic individuals who gained Medicaid coverage.

### 3.3 Health care access

We also observed varying changes in access to care (Table 2). For those gaining Medicaid, we saw a 14.6 percentage point increase in reporting a usual source of care compared to a 1.8 percentage point increase for those remaining uninsured, both of which were statistically significant. While we found significant decreases in being unable to get necessary medical care among both groups, the decrease was larger for those gaining Medicaid (8.0 percentage points) compared to those remaining uninsured (1.0 percentage point). There was a significant 4.5 percentage point decline in reporting a delay in getting necessary medical care for those gaining Medicaid, compared to a not statistically significant decline of 0.5 percentage points among those remaining uninsured. Lastly, both groups reported declines in being unable to get necessary prescription drugs—a 3.6 percentage point decline for those gaining Medicaid compared to a 0.8 percentage point decline for those remaining uninsured.

Usual source of care increased significantly among all racial/ethnic groups of Medicaid gainers, but the gains were smallest for Black, non‐Hispanic individuals (Table 3). Inability to get necessary care decreased to a similar degree across racial/ethnic groups. Only White, non‐Hispanic individuals reported significant decreases in delayed care due to costs and inability to get necessary prescriptions.

### 3.4 Health outcomes

Individuals who gained Medicaid and those remaining uninsured both reported just under a 2.5 percentage point decrease in the probability of reporting fair or poor health, although this was only statistically significant for those remaining uninsured (Table 2). Fair or poor mental health did not change significantly in either group. However, those gaining Medicaid reported a significant 4.3 percentage point decrease in severe psychological distress, while those remaining uninsured reported a small and not statistically significant decrease.

Changes in health outcomes were not significant across racial/ethnic groups gaining Medicaid, with the exception of severe psychological distress among Hispanics, which declined by 5.9 percentage points (Table 3).

### 3.5 Difference‐in‐differences estimates

In addition to examining changes in mean values of each outcome across Medicaid gainers and persistently uninsured individuals, we also obtained adjusted difference‐in‐differences estimates. For those gaining Medicaid compared to those remaining uninsured, we found significant increases in both total costs ($1612 [95% CI: $919, $2305]) and total prescription drug costs ($546 [95% CI: $290, $802]) (Table 4). Increases in total costs were only significant among White, non‐Hispanic and Hispanic individuals who gained Medicaid relative to their peers who remained uninsured. As expected, we also found those gaining Medicaid had significantly lower out‐of‐pocket costs (−$237 [95% CI: −$422, −$51]), as well as lower, but not statistically significant, out‐of‐pocket prescription drug costs (−$157 [95% CI: −$324, $11]), a finding that was generally consistent across racial/ethnic groups.

**Table 4:** Changes in health care costs, utilization, access, and outcomes among nonelderly uninsured adults who gained Medicaid

Outcome	Adjusted difference‐in‐differences estimates (95% CI)^a^			
	All	White, non‐Hispanic	Black, non‐Hispanic	Hispanic
Health care costs				
Total costs ($)^b^	1612 (919 to 2305)***	2249 (1016 to 3482)***	175 (−681 to 1031)	1437 (132 to 2742)*
Out‐of‐pocket costs ($)^b^	−237 (−422 to −51)*	−371 (−747 to 5)	−268 (−509 to −27)*	−75 (−176 to 26)
Total prescription drug costs ($)b	546 (290 to 802)***	721 (214 to 1228)**	353 (40 to 666)*	318 (106 to 530)**
Out‐of‐pocket prescription drug costs ($)^b^	−157 (−324 to 11)	−347 (−695 to 1)	−16 (−57 to 25)	−13 (−88 to 63)
Health care utilization				
Any ED visit (%)^b^	3.1 (−2.2 to 8.3)	1.2 (−7.8 to 10.3)	2.6 (−5.0 to 10.1)	3.7 (−4.7 to 12.2)
ED visits/person^b^	0.07 (−0.02 to 0.15)	0.05 (−0.11 to 0.21)	0.06 (−0.05 to 0.10)	0.08 (−0.04 to 0.20)
Any inpatient visit (%)^b^	2.3 (−0.4 to 5.1)	3.4 (−1.7 to 8.6)	0.1 (−3.8 to 4.0)	2.7 (−0.8 to 6.2)
Inpatient visits/person^b^	0.03 (−0.008 to 0.07)	0.04 (−0.02 to 0.11)	−0.01 (−0.06 to 0.5)	0.05 (−0.03 to 0.13)
Any prescription drug fill (%)^b^	12.1 (7.1 to 17.1)***	7.1 (−1.5 to 15.6)	19.2 (12.0 to 26.4)***	13.2 (5.4 to 21.0)***
Prescription drug fills/person^b^	5.7 (4.3 to 7.0)***	7.0 (4.7 to 9.3)***	5.3 (1.6 to 9.1)**	2.7 (1.4 to 4.1)***
Health care access				
Have usual source of care (%)	12.3 (7.8 to 16.8)***	11.8 (3.9 to 19.6)**	8.5 (0.0 to 17.1)	13.1 (5.0 to 21.2)**
Unable to get necessary medical care (%)	−6.4 (−10.3 to −2.4)**	−6.9 (−14.5 to 0.7)	−5.7 (−11.4 to 0.1)	−8.5 (−13.9 to −3.0)**
Delayed necessary medical care (%)	−3.7 (−7.5 to 0.1)	−6.8 (−14.2 to 0.5)	−1.1 (−6.1 to 3.9)	−2.2 (−8.2 to 3.9)
Unable to get necessary prescription (%)	−2.8 (−6.4 to 0.8)	−6.6 (−14.1 to 0.9)	3.4 (−0.6 to 7.4)	−0.1 (−4.5 to 4.3)
Health outcomes				
Fair/poor health (%)^b^	−0.2 (−4.9 to 4.5)	−2.3 (−9.7 to 5.1)	0.7 (−5.9 to 7.2)	2.2 (−4.4 to 8.8)
Fair/poor mental health (%)^b^	−2.6 (−6.3 to 1.2)	−3.9 (−11.1 to 3.2)	1.6 (−4.2 to 7.5)	−3.6 (−8.6 to 1.4)
Severe psychological distress (%)	−3.8 (−7.4 to −0.3)*	−4.1 (−10.6 to 2.4)	−3.9 (−11.0 to 3.2)	−4.9 (−9.8 to 0.0)*

ED, emergency department.

*P <* *0.05; *^*^ *P *<* *0.01; *^**^ *P *<* *0.001.

Adjusted for age, sex, race/ethnicity, marital status, education, region, family size, family income, year, and number of chronic conditions.

Based on Medical Expenditure Panel Survey rounds 1 and 2 (Period 1) and rounds 3 and 4 (Period 2).

We found no significant differences in changes in ED or inpatient utilization patterns between those gaining Medicaid and those remaining uninsured (Table 4). The only statistically significant health care utilization differences we found were that those gaining Medicaid were 12.1 percentage points more likely to have a prescription drug fill (95% CI: 7.1, 17.1) and had 5.7 more prescription drug fills (95% CI: 4.3, 7.0) between each period, compared to those who remained uninsured. There were statistically significant increases in any prescription drug fill among Black, non‐Hispanic and Hispanic individuals who gained Medicaid relative to uninsured peers, but the magnitude of gains in mean prescription fills was largest among White, non‐Hispanics who gained Medicaid.

There were relatively large differences in changes in individuals’ access to care (Table 4). We found a statistically significant 12.3 percentage point increase (95% CI: 7.8, 16.8) in reporting a usual source of care for those gaining Medicare compared to those who remained uninsured. Similarly, compared to those remaining uninsured, there was a significant 6.4 percentage point decline (95% CI: −10.3, −2.4) in reporting being unable to get necessary care. There were no significant differences in other access measures. Usual source of care increased significantly for non‐Hispanic White and Hispanic individuals who gained Medicaid relative to the remaining uninsured and did not change significantly for Black, non‐Hispanic individuals. Inability to obtain necessary medical care decreased significantly among Hispanic individuals who gained Medicaid.

Finally, we found modest changes in health outcomes (Table 4). We did not find statistically significant differences in changes in self‐reported fair/poor general or mental health. However, there was a significant 3.8 percentage point decline (95% CI: −7.4, −0.3) in severe psychological distress among Medicaid gainers overall, compared to those remaining uninsured. Within racial/ethnic groups, severe psychological distress decreased significantly only among Hispanic individuals who gained Medicaid compared to their peers who remained uninsured.

### 3.6 Sensitivity analyses

In our first sensitivity analysis, we examined linear trends in Period 1 using multivariable linear regression models. We found that trends were generally similar for individuals who gained Medicaid and those that remained uninsured. Statistically significant, though quantitatively modest, differences in linear trends during Period 1 were identified for three measures: total costs, any prescription drug fill, and total prescription drug fills (Table S1). Total cost and total prescription fill differences in Period 1 were <25 percent of our difference‐in‐differences estimate. Therefore, differences in Period 1 are unlikely to explain the large differences observed between Period 1 and Period 2. Differences in Period 1 are also illuminating and suggest individuals who gain Medicaid likely face escalating health costs that may result, through a variety of mechanisms, in enrollment in public health insurance coverage.

Our first entropy‐balanced model was weighted on our baseline set of covariates, and our second model was additionally weighted based on round 1 and 2 outcome values, which eliminated Period 1 trend differences (Table S2). Entropy‐balanced estimates were substantively similar to each other and to our primary analysis, with one exception. When we weighted on round 1 and 2 outcomes, our difference‐in‐differences estimates for increases in ED visits and inpatient visits became statistically significant, and the decrease in out‐of‐pocket spending, while similar in magnitude, was no longer statistically significant.

We also re‐estimated cost models using a two‐part model. We found smaller, though still statistically significant, increases in total costs and total prescription drug costs, as well as significant reductions in both total out‐of‐pocket costs and out‐of‐pocket prescription drug costs (Table S3); these results did not substantively alter our main findings.

In a final sensitivity analysis, we varied the definitions for both study groups. For those gaining Medicaid, we varied the length of uninsured months and time enrolled in Medicaid; for those remaining uninsured, we varied the requirements for the months remaining uninsured. Results were similar to estimates from our primary model specification (Table S4).

## 4 DISCUSSION

Among a national sample of previously uninsured Americans, enrollment in Medicaid was associated with significant increases in total medical spending and reductions in out‐of‐pocket costs, higher levels of prescription medication use, and improvements in access to care relative to individuals who remained uninsured. Those who gained Medicaid also had meaningful improvements in serious psychological distress. These findings suggest Medicaid has economically and clinically important effects on outcomes that matter to both patients and policymakers.

In unadjusted analyses of individuals who gained Medicaid, we found important differences by race/ethnicity. For example, total costs increased significantly for White, non‐Hispanic and Hispanic individuals but remained relatively flat for Black, non‐Hispanic individuals. These findings may be related to disparate access to care after Medicaid enrollment. Improvements in access to care were significant for each outcome measure among White, non‐Hispanic individuals, whereas measures of delayed medical care and obtaining necessary prescriptions were not significant for Black, non‐Hispanics and Hispanics. Further, gains in any prescription drug fill were largest among Black, non‐Hispanic and Hispanic individuals, but changes in total prescription drug costs were largest among white, non‐Hispanic individuals. Our data are consistent with population‐level analyses—while Medicaid enrollment improves care across racial and ethnic groups, Medicaid alone is unlikely to eliminate racial/ethnic disparities in access to care and health in the United States.^12^, ^21^, ^22^, ^35^

In our primary multivariable analysis, enrollment in Medicaid was not associated with statistically significant changes in ED use or inpatient hospitalizations. However, these results should be interpreted with some caution. First, in our primary model specification, Medicaid enrollment was associated with trends toward higher ED and inpatient. Second, Medicaid enrollment was associated with significantly higher levels of ED use in sensitivity models that used entropy balancing. Our primary specification is consistent with prior work that indicated expansion of Medicaid was not associated with increased ED use,^36^ but contrasts with findings from the OHIE which found higher levels of ED use after Medicaid enrollment.^17^ A recent editorial on the issue noted, “the relationship between health insurance and emergency care isn't straightforward.”^37^ Our primary analysis adds a null finding to this debate, but with the caveats noted above.

Our examination of Medicaid enrollment also aligned with the existing literature. As might be expected with an individual‐level analysis, the study identified somewhat larger effects on access to care after 1 year or less of Medicaid enrollment than prior work had observed at the population level.^38^, ^39^ For example, Wherry and Miller found a non‐significant 4.3 percentage improvement in usual source of care one year after Medicaid expansion under the ACA, whereas this study estimated a significant 12.3 percentage point improvement. The difference in our estimates is likely due to our focus specifically on individuals who enroll in Medicaid after being uninsured, whereas prior studies examined the effect of Medicaid expansion as a potential opportunity for Medicaid enrollment regardless of prior coverage status. Estimates of changes in out‐of‐pocket costs ($215 vs $237) and any prescription medication use (11.6 percentage points vs 12.1 percentage points) were very similar to those found in a randomized control trial after 2 years of Medicaid enrollment.^16^, ^18^ Finally, even after adjustment, Medicaid enrollment was associated with statistically significant reductions in severe psychological distress, a finding that is similar to previous evaluations that found Medicaid expansion under the ACA was associated with significant reductions in psychological distress, poor mental health days, and depression.^8^, ^16^, ^40^

### 4.1 Limitations

Study findings should be considered in light of limitations related to our data source and study design. First, our statistical adjustments may not fully control for selection bias regarding who enrolls in Medicaid. Specifically, somewhat asymmetric trends in Period 1 in comparisons of individuals who remain uninsured vs enroll in Medicaid suggest that uninsured individuals who enroll in Medicaid may do so for reasons related to their need for medical care. While we were unable to fully adjust for these possibilities, we employed entropy balancing in our sensitivity analyses as an additional type of control. Estimates of costs, prescription drug utilization, and access to care from our entropy balancing models were substantively similar to our primary specification.

A second limitation is that only 1 year of data was available after the ACA‐sponsored Medicaid expansion occurred in some states (i.e, 2014), and therefore, we could not determine whether gaining Medicaid coverage under the ACA had differential effects compared to gaining Medicaid in prior years. A final limitation is that the access to care outcomes refers to the preceding 12 months. While these measures were generally obtained toward the end of each MEPS data period (i.e, quarter 3 or 4), some responses might refer to previous periods of insurance and bias estimates toward the null. However, we are unaware of other contemporary datasets that could be used to track uninsured populations into Medicaid coverage to examine the outcomes presented in this study at the national level.

## 5 CONCLUSION

Twenty‐eight million Americans remain uninsured in the United States,^41^ and this number is expected to rise following the recent repeal of tax penalties related to the individual mandate and other federal changes to Medicaid eligibility.^42^, ^43^ Our study suggests Medicaid is a powerful tool for improving access to care and affordability across racial and ethnic groups and has important effects on mental health. Implementation of Medicaid expansion in states that have not yet expanded through the ACA could further reduce disparities between uninsured and insured populations, while cuts to existing Medicaid programs could threaten to reverse some of the gains made in recent years. Additional interventions, beyond Medicaid, are likely needed to reduce racial/ethnic disparities in access to care and health outcomes.

## CONFLICT OF INTEREST

None.
