# Supplementary material for: Medicaid enrollment among previously uninsured Americans and associated outcomes by race/ethnicity—United States, 2008‐2014
Source: Health Serv Res. 2018 Nov 5;54(Suppl 1):297–306. doi: 10.1111/1475-6773.13085 (PMC6341200; doi:10.1111/1475-6773.13085)
Supplement: Supplementary file 2 [file HESR-54-297-s002.docx]

**Supplemental Information**

Medicaid Enrollment among Previously Uninsured Americans and Associated Outcomes by Race/Ethnicity - United States, 2008-2014

PAGE 2 **Table S1.** Differences in Period 1 trends among individuals who gained Medicaid relative to those who remained uninsured

PAGE 3  **Table S2.** Changes in health care costs, utilization, access, and outcomes among non-elderly uninsured adults who gained Medicaid relative to those who remained uninsured using entropy weighting

PAGE 4 **Table S3.** Two-part models for highly skewed cost outcomes

PAGE 5  **Table S4.** Changes in health care costs, utilization, access, and outcomes among non-elderly uninsured adults who gained Medicaid relative to those who remained uninsured by varying months of enrollment

**Table S1.** Differences in Period 1 trends among individuals who gained Medicaid relative to those who remained uninsured

|  | **Differential trends among individuals who gained Medicaid and those who remained uninsured** | | |
| --- | --- | --- | --- |
| **Outcome** | **Coefficient** | **95% CI** | ***P* value** |
| **Health care costs** |  |  |  |
| Total costs ($) | 396 | (83-710) | 0.014 |
| Out-of-pocket costs ($) | 26 | (-143-195) | 0.761 |
| Total prescription drug costs ($) | 74 | (-112-260) | 0.434 |
| Out-of-pocket prescription drug costs ($) | 3.8 | (-153-161) | 0.962 |
| **Health care utilization** |  |  |  |
| Any ED visit (%) | 2.0 | (-1.8-5.9) | 0.295 |
| ED visits/person | 0.01 | (-0.05-0.07) | 0.758 |
| Any inpatient visit (%) | 0.3 | (-1.5-2.1) | 0.723 |
| Inpatient visits/person | 0.003 | (-0.02-0.03) | 0.821 |
| Any prescription drug fill (%) | 7.0 | (3.0-11.0) | <0.001 |
| Prescription drug fills/person | 1.4 | (0.70-2.03) | 0.001 |
| **Health care access** |  |  |  |
| Have usual source of care (%) | NA | NA | NA |
| Unable to get necessary medical care (%) | NA | NA | NA |
| Delayed necessary medical care (%) | NA | NA | NA |
| Unable to get necessary prescription (%) | NA | NA | NA |
| **Health outcomes** |  |  |  |
| Fair/poor health (%) | 0.2 | (-4.6-5.0) | 0.926 |
| Fair/poor mental health (%) | 2.2 | (-1.0-5.3) | 0.185 |
| Estimates are adjusted for age, sex, race/ethnicity, marital status, education, region, family size, family income, year, and number of chronic conditions.  NA = Not applicable. Access measures are assessed in the Medical Expenditure Panel Survey once each year of the study. | | | |

**Table S2.** Changes in health care costs, utilization, access, and outcomes among non-elderly uninsured adults who gained Medicaid relative to those who remained uninsured using entropy weighting

|  | **(A)**^b^ | | **(B)**^c^ | |
| --- | --- | --- | --- | --- |
| **Outcome** | **Entropy balancing estimate** | **95% CI** | **Entropy balancing estimate** | **95% CI** |
| **Health care costs** |  |  |  |  |
| Total costs ($)^a^ | 937*** | (406-1,468) | 1325*** | (716-1,934) |
| Out-of-pocket costs ($)^a^ | -151* | (-277- -26) | -106 | (-260-48) |
| Total prescription drug costs ($)^a^ | 368*** | (185-552) | 408*** | (210-607) |
| Out-of-pocket prescription drug costs ($)^a^ | -68 | (-144-9) | -60 | (-146-27) |
| **Health care utilization** |  |  |  |  |
| Any ED visit (%)^a^ | 3.0 | (-0.6-6.6) | 6.7*** | (3.0-10.3) |
| ED visits/person^a^ | 0.04 | (-0.02-0.11) | 0.1*** | (0.04-0.18) |
| Any inpatient visit (%)^a^ | 1.6 | (-0.5-3.7) | 3.9*** | (1.6-6.1) |
| Inpatient visits/person^a^ | 0.02 | (-0.01-0.06) | 0.06** | (0.02-0.09) |
| Any prescription drug fill (%)^a^ | 12.9*** | (8.7-17.1) | 16.8*** | (12.6-21.0) |
| Prescription drug fills/person^a^ | 3.9*** | (2.48-5.23) | 4.4*** | (2.95-5.93) |
| **Health care access** |  |  |  |  |
| Have usual source of care (%) | 13.8*** | (9.3-18.3) | 13.7*** | (9.2-18.2) |
| Unable to get necessary medical care (%) | -4.7** | (-7.7- -1.7) | -4.8** | (-7.7- -1.8) |
| Delayed necessary medical care (%) | -0.9 | (-3.5-1.7) | -0.9 | (-3.5-1.7) |
| Unable to get necessary prescription (%) | -0.6 | (-3.1-1.9) | -0.7 | (-3.2-1.8) |
| **Health outcomes** |  |  |  |  |
| Fair/poor health (%)^a^ | 1.4 | (-2.7-5.5) | 4.0 | (0.0-8.1) |
| Fair/poor mental health (%)^a^ | -2.3 | (-6.0-1.4) | 1.4 | (-2.4-5.3) |
| Severe psychological distress (%) | -3.1 | (-6.5-0.3) | -2.7 | (-6.3-0.9) |
| ^*^*P*<.05; ^**^*P*<.01; ^***^*P*<.001  ^a^Based on MEPS rounds 1 and 2 (Period 1) and rounds 3 and 4 (Period 2).  ^b^Control group weighted to match treatment based on age, sex, race/ethnicity, marital status, education, region, family size, family income, year, and number of chronic conditions, all of which were also controlled for in our primary difference-in-difference estimates from linear regression.  ^c^In addition to covariates in (A), also weighted based on round 1 and 2 values of the outcome. For outcomes without measures in both round 1 and 2, weighted using round 1 and 2 costs. This more conservative approach ensured no difference in pre-period (Period 1) trends. | | | | |

|  | BASELINE (weighted) | |  | Two-part model^a^ | |
| --- | --- | --- | --- | --- | --- |
|  | **Adjusted difference-in-differences estimate**^b^ | 95% CI |  | **Adjusted difference-in-differences estimate**^b^ | 95% CI |
| Total costs (inflation adjusted) | $1,612*** | 919, 2,304 |  | 581*** | 301, 861 |
| Total out-of-pocket costs (inflation adjusted) | -$237* | -422, -51 |  | -190** | -307, -74 |
| Total prescription costs (inflation adjusted) | $546*** | 290, 802 |  | 131** | 49, 212 |
| Total out-of-pocket prescription costs (inflation  adjusted) | -$157 | -324, 11 |  | -97** | -167, -27 |
| ^*^*P*<.05; ^**^*P*<.01; ^***^*P*<.001  ^a^The two-part model results are based on the twopm command in Stata (Belotti et al. 2015) where we use a logit model to predict having any costs and generalized linear model with a log link and gamma distribution to estimate costs for those having positive costs.  ^b^Adjusted for age, sex, race/ethnicity, marital status, education, region, family size, family income, year, and number of chronic conditions. | | | | | |

**Table S3.** Two-part models for highly skewed cost outcomes

**Reference**

Belotti, F., P. Deb, W. Manning, and E. Norton. 2015. “Twopm: Two-Part Models.” *Stata Journal*, 15(1): 3–20.

**Table S4.** Changes in health care costs, utilization, access, and outcomes among non-elderly uninsured adults who gained Medicaid relative to those who remained uninsured by varying months of enrollment

|  | **Changing treatment group**^b^ | | | | **Changing control group**^b^ | |  |
| --- | --- | --- | --- | --- | --- | --- | --- |
| **Outcome** | T1: Uninsured 3 months  T2: Medicaid 9+ months | T1: Uninsured 6+ months  T2: Medicaid 6+ months | T1: Uninsured 6+ months  T2: Medicaid 9+ months | T1: Uninsured 9 months  T2: Medicaid 6+ months | T1: Uninsured 12 months in year 1  T2: Uninsured 9+ months in year 2 | T1: Uninsured 9 months in year 1  T2: Uninsured 12 months in year 2 | |
| **Health care costs** |  |  |  |  |  |  | |
| Total costs ($)^a^ | 1,022* | 1,863*** | 1,471*** | 1,858*** | 1,597*** | 1,611*** | |
| Out-of-pocket costs ($)^a^ | -227** | -295* | -243* | -317* | -237* | -246** | |
| Total prescription drug costs ($)^a^ | 559*** | 499** | 548*** | 519** | 547*** | 543*** | |
| Out-of-pocket prescription drug costs ($)^a^ | -133 | -232 | -157 | -242 | -156 | -161 | |
| **Health care utilization** |  |  |  |  |  |  | |
| Any ED visit (%)^a^ | 0.6 | 5.4 | 2.5 | 5.0 | 2.9 | 3.0 | |
| ED visits/person^a^ | 0.02 | 0.10 | 0.08 | 0.10 | 0.06 | 0.06 | |
| Any inpatient visit (%)^a^ | -0.6 | 3.0 | 1.7 | 3.2 | 2.3 | 2.3 | |
| Inpatient visits/person^a^ | -0.01 | 0.05 | 0.03 | 0.05 | 0.03 | 0.03 | |
| Any prescription drug fill (%)^a^ | 11.1*** | 13.8*** | 13.7*** | 14.1*** | 12.1*** | 12.3*** | |
| Prescription drug fills/person^a^ | 6.4*** | 5.5*** | 6.5*** | 5.4*** | 5.7*** | 5.6*** | |
| **Health care access** |  |  |  |  |  |  | |
| Have usual source of care (%) | 11.5*** | 14.3*** | 14.2*** | 15.1*** | 12.3*** | 12.3*** | |
| Unable to get necessary medical care (%) | -8.8*** | -4.9 | -8.8*** | -6.9** | -6.2** | -6.3** | |
| Delayed necessary medical care (%) | -7.0*** | -1.5 | -7.6*** | -2.9 | -3.5 | -3.7 | |
| Unable to get necessary prescription (%) | -2.6 | -1.6 | -3.6 | -1.6 | -2.8 | -2.7 | |
| **Health outcomes** |  |  |  |  |  |  | |
| Fair/poor health (%)^a^ | 0.4 | -0.8 | 0.6 | -0.3 | -0.1 | -0.3 | |
| Fair/poor mental health (%)^a^ | -4.5* | -0.4 | -4.9* | -0.6 | -2.6 | -2.6 | |
| Severe psychological distress (%) | -2.0 | -3.0 | -3.8 | -2.6 | -3.9* | -3.8* | |
| ^*^*P*<.05; ^**^*P*<.01; ^***^*P*<.001  ^a^Based on MEPS rounds 1 and 2 (Period 1) and rounds 3 and 4 (Period 2).  ^b^Adjusted for age, sex, race/ethnicity, marital status, education, region, family size, family income, year, and number of chronic conditions. | | | | | | | |
